# Pervasive Lying Posture Tracking

**DOI:** 10.3390/s20205953

**Published:** 2020-10-21

**Authors:** Parastoo Alinia, Ali Samadani, Mladen Milosevic, Hassan Ghasemzadeh, Saman Parvaneh

**Affiliations:** 1 Philips Research North America, Cambridge, MA 02141, USA; parastoo.alinia@wsu.edu (P.A.); ali.samadani@philips.com (A.S.); mladen.milosevic@philips.com (M.M.); 2 School of Electrical Engineering and Computer Science, Washington State University, Pullman, WA 99164, USA; hassan.ghasemzadeh@wsu.edu

**Keywords:** lying posture tracking, traditional machine learning, ensemble classification, deep recurrent neural network models, long short-term memory sequence classification model

## Abstract

Automated lying-posture tracking is important in preventing bed-related disorders, such as pressure injuries, sleep apnea, and lower-back pain. Prior research studied in-bed lying posture tracking using sensors of different modalities (e.g., accelerometer and pressure sensors). However, there remain significant gaps in research regarding how to design efficient in-bed lying posture tracking systems. These gaps can be articulated through several research questions, as follows. First, can we design a single-sensor, pervasive, and inexpensive system that can accurately detect lying postures? Second, what computational models are most effective in the accurate detection of lying postures? Finally, what physical configuration of the sensor system is most effective for lying posture tracking? To answer these important research questions, in this article we propose a comprehensive approach for designing a sensor system that uses a single accelerometer along with machine learning algorithms for in-bed lying posture classification. We design two categories of machine learning algorithms based on deep learning and traditional classification with handcrafted features to detect lying postures. We also investigate what wearing sites are the most effective in the accurate detection of lying postures. We extensively evaluate the performance of the proposed algorithms on nine different body locations and four human lying postures using two datasets. Our results show that a system with a single accelerometer can be used with either deep learning or traditional classifiers to accurately detect lying postures. The best models in our approach achieve an $F_{1}$ score that ranges from 95.2% to 97.8% with a coefficient of variation from 0.03 to 0.05. The results also identify the thighs and chest as the most salient body sites for lying posture tracking. Our findings in this article suggest that, because accelerometers are ubiquitous and inexpensive sensors, they can be a viable source of information for pervasive monitoring of in-bed postures.

## 1. Introduction

Keeping track of in-bed lying postures and the transitions between them provide useful clinical information regarding the patients’ mobility [1,2], risk of developing hospital-acquired pressure injuries [3], hidden death in epilepsy [4], experiencing obstructive sleep apnea, circadian rhythm disorders [5], risk of sudden infant death [6], and quality of sleep [7]. The in-bed posture of the patients, especially in hospitals, is usually monitored manually and through visual observation, which is a labor- and cost-intensive task. Therefore, researchers have proposed monitoring in-bed postures continuously and non-obtrusively while using wearable sensors. Two specific medical applications of objective and continuous monitoring of in-bed posture tracking using wearable sensors are: (a) pressure injury risk assessment and prevention, (b) positional sleep apnea therapy. Prolonged lying on a specific posture can increase the risk of pressure injuries in people with compromised mobility and bed-bound patients [8]. Pressure injuries are a significant, yet potentially preventable event, and the proposed approach for pressure injury prevention is the regular repositioning of patients (e.g., repositioning every 2 h). Objective lying posture tracking using a wearable could enable its integration as a new variable in a continuous pressure injury risk assessment. It could also be used to make informed decisions regarding patient repositioning and help to assure compliance with repositioning guidelines. Sleeping posture influences the frequency of apneas and hypopneas in 50–60% of individuals with obstructive sleep apnea [9]. Positional sleep apnea therapy can reduce apnea/hypopnea episodes by preventing sleeping in a lying posture. A shirt with a foam or a tennis ball on the backside is a common solution for positional sleep apnea therapy that prevents sleeping in a supine position. Although sensor-based solutions are available for positional sleep apnea therapy (e.g., Philips NightBalance), improvement in lying posture tracking when considering both algorithm and wearable sensor positioning can reduce false alarms and improve user acceptance. Automatic in-bed lying posture tracking systems have been developed using data that were collected from sensors of different modalities, such as accelerometers [10,11,12], load cell sensors [13], pressure sensors [14,15], infrared cameras [16], electrocardiogram waveforms [17], and multi-modal systems [18,19,20]. Pressure mats and load sensor systems impose a high cost to the end-users and often require calibration. The camera-based systems usually encounter setup and privacy issues from the end-users and are more difficult to analyze than the wearable sensors [21]. Other works have utilized multiple wearable sensors on different body locations for continuous lying-posture detection, which imposes discomfort to the end-users and impedes long-term monitoring. To address these issues, we develop a traditional machine learning (ML) model for lying posture detection while using a single accelerometer sensor, which is is an ensemble of decision tree classifiers with hand-engineered time-domain features.

Deep learning (DL) has emerged as the leading approach in the field of computer vision, voice recognition, and natural language processing in recent years. Deep neural networks are known as learners of high-level features for a specific problem domain. This makes DL models suitable models for human posture estimation. Moreover, DL models tend to reduce the overhead of feature engineering compared to traditional machine learning models [22]. To date, no studies have explored the possibility of utilizing deep neural networks for acceleration-based lying posture tracking. We develop a deep learning model for lying posture detection while using a single accelerometer sensor to investigate the possibility of replacing traditional feature-based machine learning models with deep neural networks; therefore, reducing the burden of feature-engineering. Our deep learning model, adaptive long short-term memory network (AdaLSTM), is a long short-term memory network (LSTM) that uses an adaptive learning rate method with a decaying learning rate schedule.

More specific contributions of this paper are as follows. We (1) investigate the efficacy of a single accelerometer for lying-posture tracking while using feature engineered machine learning models and deep LSTM networks; (2) identify the set of optimal time-domain features for accurate lying posture detection using traditional ML; (3) compare traditional machine learning with deep learning in recognition of lying postures; (4) evaluate nine different body sites to determine the most appropriate site to attach the accelerometer for accurate lying posture tracking.

## 2. Background & Related Studies

Human posture detection is an active research area. There have been many studies to explored models to distinguish human lying and sitting postures [23,24,25]. In this section, we discuss the previous studies on lying posture tracking while using wearable accelerometer sensors. We divide these studies based on the number of wearable sensors into (1) multi-sensor and (2) single-sensor lying posture tracking.

### 2.1. Multi-Sensor Lying Posture Tracking

A study in [12] conducted by Kwasnicki et al. proposed a lightweight sensing platform for monitoring sleep quality and posture using three wearable accelerometer sensors that were placed on both arms and the chest. They applied a K-nearest neighbor, naive Bayes, and decision tree classifiers on the mean and variance of each axis of the signal from all three accelerometer sensors. Their models achieved 99.5% average accuracy in detecting the four major lying postures (i.e., lying supine, prone, and laterals). In another study fallmann et al. proposed a lying posture detection algorithm using three accelerometer sensors on the chest and the legs. Their algorithm first, classified the postures using the acceleration-moving variance method, into stable and non-stable time windows, then classified the features into the postures prone, supine, and laterals. Their model achieved an average accuracy of 83.6% [26]. Moreover, Wrzus et al. [10] developed a 99.7% accurate classification model using chest and thigh accelerometry data based on the angular orientation of the upper body along the vertical axis to classify lying postures.

The above-mentioned algorithms that rely on data from multiple sensors attached to the different locations on the user’s body cause discomfort and limit usability, especially for long-term monitoring during sleep. Moreover, the possibility of sensor rotation during sleep might alter the angular axes of the sensors relative to each other, therefore decrease the accuracy of the orientation-based lying posture tracking. In this paper, we proposed lying posture detection algorithms that only use data from a single accelerometer sensor, which can be placed on one of the nine different body locations, including chest, thighs, ankles, arms, and wrists. Therefore, the proposed models using a single accelerometer are more comfortable to the end-users are favored over those using multi-sensor.

### 2.2. Single-Sensor Lying Posture Tracking

Monitoring lying postures while using a single sensor improves the usability and wearability of the system comparing to the multi-sensor framework. These advantages in single-sensor monitoring become more evident in older adults and users with cognitive impairment, who might face difficulties to recall and follow the protocols [27]. Moreover, single-sensor lying posture tracking is preferred by the clinical team and caregivers of hospitalized patients, since these patients are usually required to be connected to several monitoring devices and IV lines [28].

In a study conducted by Razjouyan et al. in [29], the authors developed a lying posture detection algorithm based on a single accelerometer sensor on the chest of the user. They used a logistic regression model on 43 time-domain features that were extracted from the magnitude of tri-axial accelerometer signal. The proposed model achieved 87.8% accuracy in detecting the lying postures supine, prone, and laterals for 21 users. In another study [11] by Zhang et al., the authors assessed the possibility of using a single accelerometer sensor on the chest to detect the lying posture during sleep. They used linear discriminant analysis (LDA) classifier on the mean value of each axis of the acceleration signal. They achieved an overall accuracy of 99% for classifying lying postures (lying supine, prone, and laterals). However, the authors of this study did not assess the effect of sensor location on the accuracy of lying posture tracking. In another study, Chang et al. developed a system that captured information regarding sleep events using a smartwatch. Their system distinguished sleep postures supine, prone, and laterals at an average precision of 96%. Their proposed algorithm detected the sleeping postures by combining the position of both hands and classification of features using a template-based Euclidean distance matching approach [30]. However, the performance of such a model is highly dependent on the quality of pre-defined hand positions and sleep posture templates. Furthermore, the possible sensor rotations during sleeping might affect the accuracy of hand position recognition; and therefore affect the lying posture detection. Moreover, Jeng et al. [31] proposed a sleep position detection algorithm that achieved 90% accuracy in detecting postures supine, prone, and laterals while using the data that were collected from an accelerometer sensor on the wrist of the users. Their proposed model applied a support vector machine classifier with a linear kernel and a random forest of 100 trees on the mean value of the signal.

## 3. Methodologies

**Figure 1:**
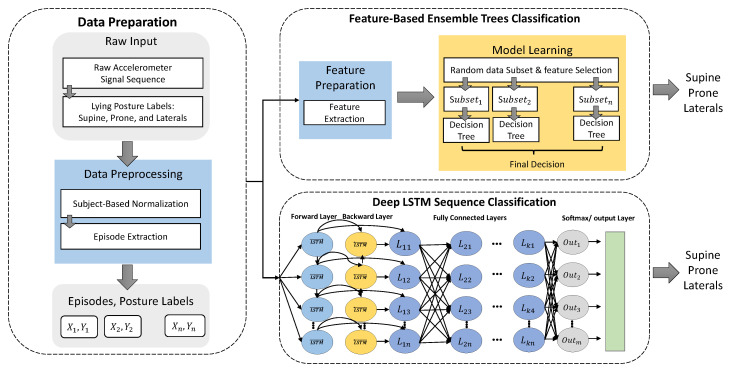
The process of training feature-based ensemble trees and Deep LSTM classifiers. Symbol $L_{kn}$ represents the fully connected unit *n* at layer *k*. Symbol *k* refers to the number of the fully connected layers and symbol *m* shows the number of the output classes.

Figure 1 shows the overall architecture of the proposed ML and DL lying posture tracking. The overall training process includes two steps of data preparation and model development.

### 3.1. Data Preparation

We define an episode of data as a sequence of signals collected from one subject while performing a run of a specific posture (e.g., lying supine). The raw accelerometer signal and lying posture labels are fed into the data processing unit. The extracted episodes consist of four major lying postures, such as lying on the back (supine), lying on the front (prone), and lying on sides (right and left laterals) that are more common across hospitalized patients and healthy individuals. The processing unit normalizes the signal to remove possible subject-based variations and segments it into different lying posture episodes based on the labels.

### 3.2. Traditional Lying Posture Tracking

The proposed traditional lying posture tracking consists of two main steps: (1) feature preparation in which an exhaustive set of features are extracted from each input episode; and, (2) ensemble model learning, which trains an ensemble of 100 decision trees on the features and lying posture labels.

#### 3.2.1. Feature Preparation

We extract 48 time-domain features (16 features from each axis of the accelerometer signal) from a sliding window over each episode of data (lying supine, prone, and on the left side) with a 50% overlap. We set the window size to the minimum episode length in the training dataset (e.g., 96 samples equal to 3.8 s). The selected features, shown in Table 1, are proven to be useful in human posture and activity recognition applications, such as amplitude, mean, standard deviation, and angle of the signal [32,33]. The three indices for each feature in Table 1 refer to features extracted from three axes of the accelerometer signal. We compute a set of meta-features $f_{i}=\left\{f_{i}^{1},f_{i}^{2},\ldots ,f_{i}^{48}\right\}$ for *i*th episode by averaging all of the extracted features from multiple windows over that episode.

#### 3.2.2. Ensemble Model Learning

A total of 48 features are obtained from the previous step. We train an ensemble of 100 decision trees on the features and lying posture labels. We choose the bagging technique as the ensemble method for reducing the variance of the decision tree and overfitting to the existing data. A decision tree is selected as the weak learner because of the high dimension of the input features. As shown in Figure 1, we fit 100 decision trees on 100 random subsets of the original dataset with a randomly selected subset of features in order to minimize the correlation between individual trees. The final prediction is the majority voting on the decision of the individual trees [34]. This model is also referred to as a random forest classifier [35].

### 3.3. Deep Lying Posture Tracking

Recurrent Neural Networks (RNNs) are a type of deep learners that are well-suited to model sequential data. However, RNNs fail to learn long-term dependencies in the data due to the problem of vanishing/exploding gradients. The Long Short-term Memory (LSTM) network has been introduced to address this issue and capture long-term dependencies from the sequential time series data [36]. LSTM networks have shown promising results on time series classification tasks [37,38]. LSTM captures long-distance dependencies from sequential data through the integration of memory cells and RRNs [39]. Bidirectional long short-term memory (bi-LSTM) networks were introduced as an extension to the LSTM networks. The bi-LSTM architecture consists of two LSTMs that train in two directions; therefore, it is capable of extracting long-term data dependencies in both forward and backward directions and learn from both past and future data [40].

At each time-step, *t*, a bi-directional LSTM network maintains two hidden layers, one for the forward propagation and another for the backward propagation. At time-step *t*, each intermediate LSTM unit from layer (i) receives the hidden state of the previous layer at the current time-stamp $h_{t}^{\left(i-1\right)}$, and state of the same layer at the previous time-step $h_{t-1}^{\left(i\right)}$ in forward propagation and next time-step $h_{t+1}^{\left(i\right)}$ in the backward propagation, as shown in Equations (1) to (4). Both forward hidden sequence, $\vec{h_{t}}$, and backward hidden sequence $\overset{\leftarrow }{h_{t}}$ are computed independently based on Equations (1) and (2). The final hidden state sequence $h_{t}$ is computed by combining the forward and backward hidden state sequences in Equation (3). The final classification result, $\hat{y}$, is combination of the results that are produced by both forward and backward hidden layers, as shown in Equations (3) and (4).

(1) $${\vec{h}}_{t}^{\left(i\right)}=f\left({\vec{W}}^{\left(i\right)}{\vec{h}}_{t}^{\left(i-1\right)}+{\vec{V}}^{\left(i\right)}{\vec{h}}_{t-1}^{\left(i\right)}+{\vec{b}}^{\left(i\right)}\right)$$

(2) $${\overset{\leftarrow }{h}}_{t}^{\left(i\right)}=f\left({\overset{\leftarrow }{W}}^{\left(i\right)}{\overset{\leftarrow }{h}}_{t}^{\left(i-1\right)}+{\overset{\leftarrow }{V}}^{\left(i\right)}{\overset{\leftarrow }{h}}_{t+1}^{\left(i\right)}+{\overset{\leftarrow }{b}}^{\left(i\right)}\right)$$

(3) $$h_{t}=U\left[{\vec{h}}_{t}^{\left(L\right)};{\overset{\leftarrow }{h}}_{t}^{\left(L\right)}\right]$$

(4) $${\hat{y}}_{t}=g\left(h_{t}+c\right)$$

At each layer (i), ${\vec{W}}^{\left(i\right)}$ and ${\overset{\leftarrow }{W}}^{\left(i\right)}$, ${\vec{V}}^{\left(i\right)}$, and ${\overset{\leftarrow }{V}}^{\left(i\right)}$ are matrices of scalar weight values between 0 and 1 corresponding to ${\vec{h}}_{t}^{\left(i-1\right)}$, ${\overset{\leftarrow }{h}}_{t}^{\left(i-1\right)}$, ${\vec{h}}_{t-1}^{\left(i\right)}$, ${\overset{\leftarrow }{h}}_{t+1}^{\left(i\right)}$, respectively. Symbols ${\vec{b}}^{\left(i\right)}$ and ${\overset{\leftarrow }{b}}^{\left(i\right)}$ refer to the bias vectors of scalar values for the forward and backward propagation, respectively. The symbol *U* is the weight matrix that corresponds to $h_{t}$. Symbols *f* and *g* are the activation function that is applied to the output of each LSTM unit (e.g., sigmoid function) and the final hidden state $h_{t}$, respectively.

In the next section, we define lying posture prediction from the sequences of raw sensor data as an optimization problem, and then design a deep learning architecture using Bi-LSTM networks as the solution.

Problem: we have *N* sequences of variable lengths where each sequence $X_{i}$ is assigned a label $Y_{i}=\left(y_{i1},y_{i2},\ldots ,yik\right)$ using max-likelihood classification, where $y_{ij}$ shows the likelihood of *j*th class for *i*th sequence. Given these, the problem is to estimate a set of labels $\hat{Y}=\left\{\hat{y_{1}},\hat{y_{2}},\ldots ,\hat{y_{N}}\right\}$, such that the difference between the actual and estimated label sets is minimized. We compute this difference as the cross-entropy of the estimated labels $\hat{Y}$ and actual label *Y* for summed over the sequences in Equation (5). Because the input sequences might adopt different length, we use a set of scalar weights $M=\{m_{1},m_{2},\ldots ,m_{i}\}$, where $m_{i}$ is the length of sequence $X_{i}$, to penalize the error that is based on the length of the sequence.

(5) $$-\sum_{i=1}^{N}\sum_{j=1}^{K}m_{i}y_{ij}\log\hat{y_{ij}}$$

where the error is a weighted (e.g., length of sequences) sum of the cross-entropy between the actual and estimated labels. The objective of the sequence classification model is to minimize Equation (5).

Deep Learning Architecture: to solve this problem, we design an Adaptive LSTM (AdaLSTM), an LSTM Network with an adaptive learning rate method with a decaying learning rate schedule. AdaLSTM receives the sequences of raw accelerometer sensor data as the input and estimates one label for each sequence. As shown in Figure 1, the input episodes/sequences of raw accelerometer data are fed to a Bi-LSTM layer with ten hidden units. The training process of the bi-LSTM includes back-propagation processes in two directions in order to minimize the error. Three fully connected layers multiply the output of the Bi-LSTM layer (e.g., a sequence of tri-axial accelerometer data) by the matrix of weights and add it by the vector of bias. The output of the fully connected layer is fed to a softmax layer that is a multi-class generalization of the logistic sigmoid function. We compute the cross-entropy loss for multi-class classification that is based on the likelihood of the softmax function. We set the maximum number of the epochs equal to 100. We set the initial learning rate and decay rate of the squared gradient moving average to 0.01 and 0.99, respectively. To shorten the amount of padding in the mini-batches and make the training more suitable for CPU, we chose the mini-batches to be short sequences of size 27. The Adam optimizer [41] is used for training the neural network through backpropagation.

## 4. Experimental Evaluation

### 4.1. Datasets & Preprocessing

We perform training and validation of the models on two publicly available datasets: (1) Class-Act dataset [42], which contains three major lying postures, including supine, prone, and left side from 22 subjects, and (2) Daily & Sports Activities Dataset (DAS) [43] that contain two major lying postures, including supine and right side from eight participants.

#### 4.1.1. Class-Act: Datasets from a Human Posture/Activity Classification

**Figure 2:**
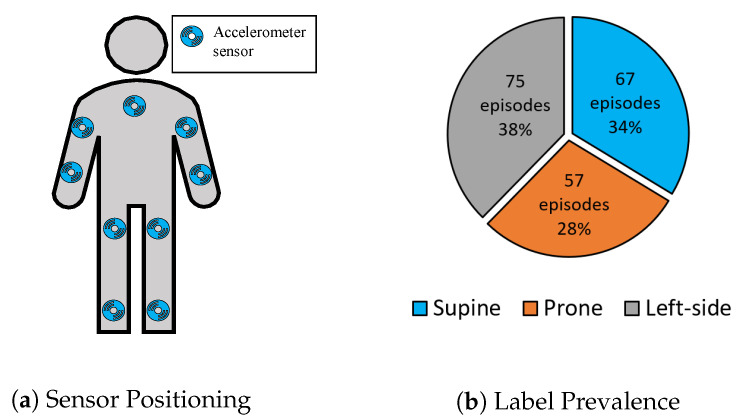
(**a**) Visualization of accelerometer sensor positioning, and (**b**) activity prevalence for Class-Act dataset.

Class-Act is a human posture and activity classification dataset from 22 healthy participants (seven females and 15 males, ages between 20 and 36) [42]. The participants wore nine accelerometer sensors that were sampled at 30 Hz on nine different body locations, including the chest, left and right thigh, left and right ankle, left and right arm, left and right wrist during the activities, as shown in Figure 2a. Class-Act was collected based on three pre-defined protocols with different combinations of activities or postures in a controlled manner. The activities were walking, sitting, standing, lying supine, lying prone, lying on the left side, kneeling, and crawling. Figure 2b shows the prevalence of the extracted episodes for only lying postures. The duration of different episodes for lying supine, prone, and on the left side were 12.2 ± 3.6, 11.9 ± 3.6, and 12.10 ± 3.3 s, respectively.

#### 4.1.2. Daily and Sports Activities Dataset (Das)

The DAS dataset contains data from eight subjects (four females and four males, ages between 20 and 30) that performed 19 activities of daily living for five minutes each [43]. The participants wore five inertial sensor units embedding a tri-axial accelerometer on the chest, right and left wrist, and right and left thigh. The sensors were calibrated in order to acquire data at a sampling frequency of 25 Hz. We only used lying supine and lying on the right side posturea in this study. The dataset contains eight episodes of lying supine and eight episodes of lying on the right side. Each episode has a length of 7500 samples (300 s).

#### 4.1.3. Integrated Dataset

**Figure 3:**
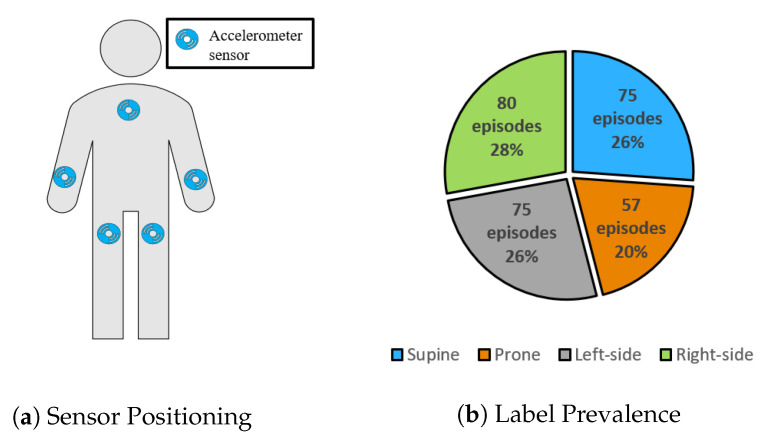
(**a**) Visualization of accelerometer sensor positioning, and (**b**) activity prevalence for the integrated dataset.

We combined the lying posture episodes for the shared sensor locations (i.e., chest, right and left wrists, and right and left thighs, as shown in Figure 3a) between the Class-Act and DAS datasets in order to validate the models in distinguishing between the four major lying postures including lying on the back, front, right side, and left side. Prior to the combination, we segmented each episode of lying supine and lying on the right side from the DAS dataset (length of 300 s) into 15 episodes of 20 s to be consistent with the average episode length from the Class-Act dataset. The result of this step was 120 episodes of lying supine and 120 episodes of lying on the right side. In the second step, to create a relatively balanced dataset, we randomly selected 80 episodes of lying on the right side and eight episodes of lying supine from the episodes of the DAS dataset that were extracted in the previous step. Finally, we combined the episodes of lying on the right side and lying supine from the DAS dataset with all of the lying episodes from the Class-Act dataset. As shown in Figure 3b, the integrated data set contained 75 episodes of supine (i.e., 26.1%), 57 episodes of prone (i.e., 19.8%), 75 episodes of the left side (i.e., 26.1%), and 80 episodes of the right side (i.e., 27.8%) for the chest, right and left wrists, and right and left thighs.

### 4.2. Comparison Metrics and Implementation Details

We validated the proposed models based on leave-one-subject-out (LOSO) validation in order to minimize overfitting to a specific subject or a specific pattern of performing a lying posture. We report accuracy, $F_{1}$ score, and balanced accuracy as evaluation metrics of the proposed models. Moreover, we perform the coefficient of variation (CoV) analysis [44] to compare the proposed algorithms against the state-of-the-art.

*Accuracy* is defined as the average effectiveness of the classifier over all the class labels.

(6) $$Accuracy=\frac{\sum_{i=1}^{l}\frac{{TP}_{i}+{TN}_{i}}{{TP}_{i}+{TN}_{i}+{FP}_{i}+{FN}_{i}}}{l}$$

where *l* is the number of class labels. $F_{1}$ score is defined as the harmonic average between *Precision* and *Recall*.

(7) $$F_{1}=\frac{2\times (Recall\times Precision)}{Recall+Precision}$$

where *Precision* refers to the average agreement of the actual class labels and classifier-predicted labels and *Recall* is the average effectiveness of the classifier to identify each class label. *Precision* and *Recall* are computed by the following equations.

(8) $$Precision=\frac{\sum_{i=1}^{l}\frac{{TP}_{i}}{{TP}_{i}+{FP}_{i}}}{l},Recall=\frac{\sum_{i=1}^{l}\frac{{TP}_{i}}{{TP}_{i}+{FN}_{i}}}{l}$$

*BalancedAccuracy* is defined as the average of the true positives and true negatives for each class label.

(9) $$BalancedAccuracy=\frac{\sum_{i=1}^{l}\frac{{TP}_{i}}{P_{i}}+\sum_{i=1}^{l}\frac{{TN}_{i}}{N_{i}}}{2\times l}$$

where l refers to the number of classes in the classification task. For each class *i* (e.g, lying supine), $P_{i}$ is the number of samples with positive label, $N_{i}$ is the number of the samples with negative labels (i.e., all classes other than the positive class lying supine), ${TP}_{i}$ refers to the positive samples that are correctly classified as belonging to class *i*, while ${FP}_{i}$ refers to the negative samples that are incorrectly classified as belonging to class *i*. ${TN}_{i}$ refers to the negative samples that are correctly classified as belonging to negative class and ${FN}_{i}$ refers to the positive samples that are incorrectly classified as belonging to negative class [45,46].

The coefficient of variation (CoV) analysis [44] is performed for each model over different body locations that are based on the equation below.

(10) $$CoV=\frac{\sigma }{\mu }$$

where σ and μ are respectively the standard deviation and average of the $F_{1}$ score in lying posture detection over different folds.

## 5. Results

In this section, we extensively evaluate the performance of the ensemble tree and AdaLSTM classifiers independently and against each other. We report the validation metrics, including accuracy, $F_{1}$ score, and balanced accuracy for both of the classification models. We further perform a coefficient of variation (CoV) analysis in order to compare the performance of the models against the state-of-the-art.

### 5.1. Raw Data Inspection

Before our main analysis, we investigate the variations in the pattern of the raw accelerometer sensor data across different subjects and different lying postures on the Class-Act dataset. We visually inspect the data by computing the mean and standard deviation of the acceleration data over all the episodes of data that were collected from different subjects while maintaining different lying postures.

**Figure 4:**
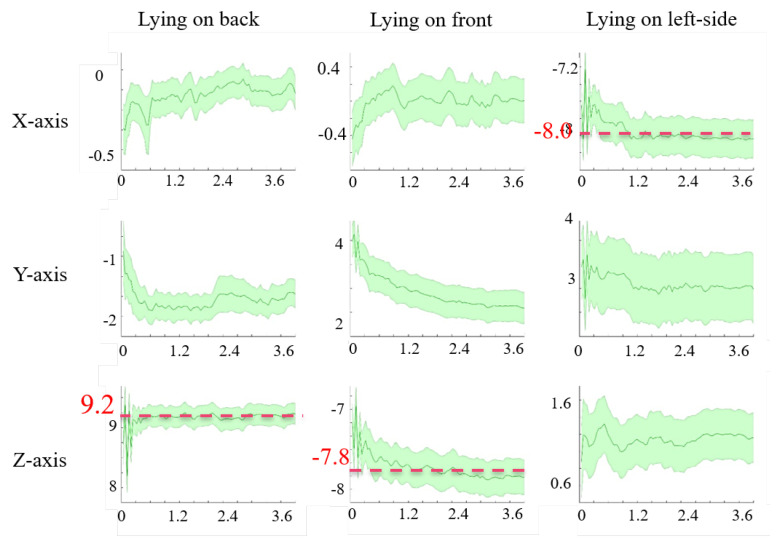
Mean and standard deviation of the magnitude of the accelerometer sensor data for different lying postures over all the subjects.

These patterns show two issues with the Class-Act dataset: (1) the accelerometer data captured from one of the subjects was not converted to gravity (g) and was stored in analog format. (2) The data collectors labeled multiple episodes of different lying posture as the same posture for one of the subjects. Figure 4 visualizes the changes in three-axis acceleration for each lying posture on the Class-Act dataset after resolving the issues that are mentioned above (e.g., correcting sensor output and labels). The solid line demonstrates the mean, and the shaded area shows the standard deviation for different episodes in a specific lying posture. The results show that the y-axis (vertical) always reports values that are near 0 g. In contrast, the x-axis (lateral) shows a mean value near −8.0 g for lying on left side posture, while it almost always reports a mean near 0 g for the other two postures. Moreover, the z-axis (horizontal) reports the mean accelerations around +9.2 g and −7.8 g for supine and prone postures, respectively, but 0g for lateral posture. Therefore, that lateral axis (x-axis) appears to be sensitive to the lying on side posture, and the horizontal axis (z-axis) appears to be sensitive to the supine and prone postures. These numbers can be justified, because, while the user is lying on one-side, the x-axis of the accelerometer sensor on the chest is parallel to gravity, therefore reporting values around g (±10). The same result occurs when the user lies on the back (supine) or front (prone), except the z-axis, becomes parallel to the g, and reports values near ±10 g. These values could be negative or positive, depending on the direction of the body (supine, prone, and lateral lying postures). Consequently, we expect these two axes to be more informative in classification compared with the y-axis.

### 5.2. Traditional Machine Learning

In this section, we validate the feature-based ensemble tree classifier in detecting three major lying postures (supine, prone, and left side) using the Class-Act dataset, including 12 subjects and nine sensor locations.

#### 5.2.1. Feature Engineering

**Figure 5:**
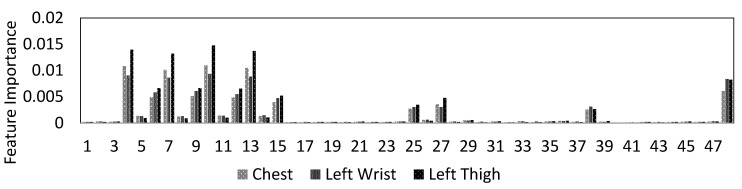
Importance of the extracted features from sensor data for lying posture tracking.

Figure 5 shows the feature importance of lying posture tracking, as determined by the ensemble tree classifier that is trained on the data from the chest, left thigh, and wrist sensors. The importance of the features is one of the outputs of the ensemble tree classification. The y-axis in this figure is the estimation of feature importance from the ensemble tree by summing over the changes in the mean squared error because of splits on every feature and dividing the sum by the number of the branch nodes in the tree [47]. The x-axis shows features 1 to feature 48 as in Table 1. Based on the results, features 4, 7, 10, and 13 are the sets with the highest importance. Table 1 shows that these features are the median, mean, maximum, and minimum of the vertical axis (e.g., x-axis) of the tri-axial accelerometer sensor. Moreover, features 6, 9, 12, and 15 are the second important set of features. Based on Table 1. These predictors refer to features median, mean, maximum, and minimum of the z-axis of the tri-axial accelerometer signal. These results match the observations on the sensitivity of the lying on the left side to the x-axis and lying supine and lying prone postures to the frontal axis (e.g., z-axis) in Figure 4.

#### 5.2.2. Lying Posture Detection

Table 2 reports the average and standard deviation of accuracy, balanced accuracy, and $F_{1}$ score of lying posture detection using the ensemble tree classifier using leave-one-subject-out (LOSO) validation. Overall, the body locations, such as the chest and the thigh, which are less susceptible to nocturnal movement during sleep, demonstrate high performance in lying posture detection, while sensor locations such as the arms and the wrists are poor in lying posture detection. More specifically, the ensemble tree classifiers trained on the data collected from the chest, thighs, or ankles achieve 89.8–96.2% accuracy, 82.9–94.4% balanced accuracy, and 82.8–93.6% $F_{1}$ score. While these values drop when the sensor is placed on the upper body parts, such as the arms and the wrists (78.6–89.5% accuracy, 62.9–84.0% balanced accuracy, and 60.9–81.6% $F_{1}$ score).

**Figure 6:**
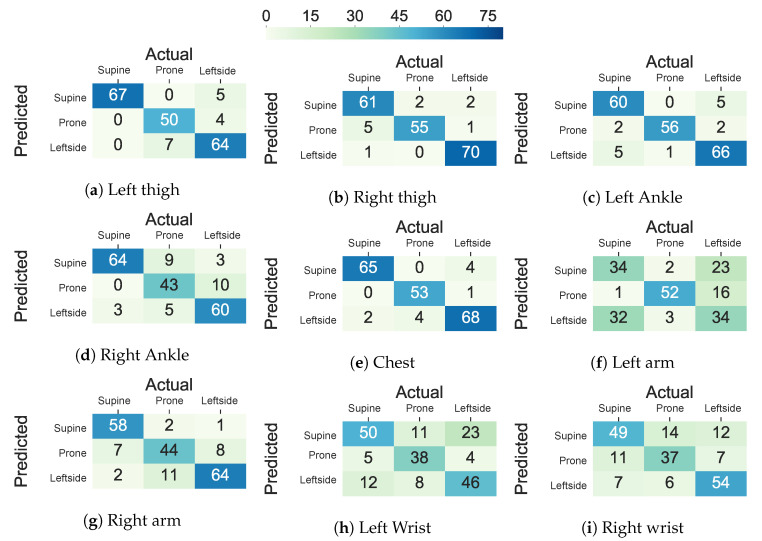
Confusion matrix of the ensemble tree classifier in classifying lying postures into supine, prone, and left side for the thighs, ankles, chest, arms, and wrists locations using Class-Act dataset.

The performance decline in the upper body parts originated from inter-subject variations in the placement of the arms and the wrists and nocturnal movements of them, such as bending and rotating while maintaining the same lying posture. The trend in the standard deviation of accuracy, balanced accuracy, and $F_{1}$ score across different subjects is also in concordance with the hypothesis that more within-subject variation (e.g., 11.7% to 23.8% standard deviation in accuracy, balanced accuracy, and $F_{1}$ score) is observed when the sensor is worn on the wrists and the arms when comparing to the chest, thighs, and ankles (e.g., 6.9% to 22.7% standard deviation in accuracy, balanced accuracy, and $F_{1}$ score). Moreover, Figure 6 visualizes the confusion matrix of lying posture classification using an ensemble tree classifier on the Class-Act dataset. The confusion matrices for the classifying the thighs, chest, and ankles data show more promising results than the arms and wrists. In particular, the classifiers trained on the left thigh, right thigh, and chest misclassify 8.1%, 5.6%, and 5.6% of the lying episodes. The misclassification rate increases to 7.6% and 39.0% for the left ankle and left arm locations, and 15.2%, 15.7%, and 28.9% for the right ankle, right arm, and right wrist locations.

### 5.3. Deep Sequence Learning

In this section, we evaluate the performance of the AdaLSTM classifier in detecting three major lying postures. Specifically, we compare the performance of the trained model on nine different on-body locations from the Class-Act dataset.

Table 3 shows the mean and standard deviation of accuracy, balanced accuracy, and $F_{1}$ score of the model while using the Class-Act dataset, including nine sensor locations. AdaLSTM achieves 94.5–98.9% average Accuracy, 92.4–98.4% average balanced accuracy, and 91.5–98.2% average $F_{1}$ score when applied to the data that were collected from the sensor worn on the chest, thighs, or ankles. However, the performance drops to 64.8–86.8% average accuracy, 64.9–79.0% average balanced accuracy, and 62.9–75.7% average $F_{1}$ score, for the cases where the sensor was on the arms and wrists. The within-subject standard deviation in the accuracy, balanced accuracy, and $F_{1}$ score is higher when the sensor is placed on the arms and the wrists (9.2–24.8%) when comparing to the thighs and chest (5.2–15.6%). Such results could be justified according to the findings from a study by Skarpsno et al., which showed the duration of nocturnal movements while sleeping in the arms and upper back was higher than the thighs in 2107 subjects [48]. AdaLSTM is the most accurate when applied to the data collected from the sensor on the left thigh (98.9% ± 8.2 accuracy, 98.4% ± 5.2 balanced accuracy, and 98.2% ± 6.2 $F_{1}$ score). The model on the left wrist achieves the lowest performance (64.8% ± 22.9% accuracy, 64.9% ± 24.8% balanced accuracy, and 62.9% ± 23.2% $F_{1}$ score).

**Figure 7:**
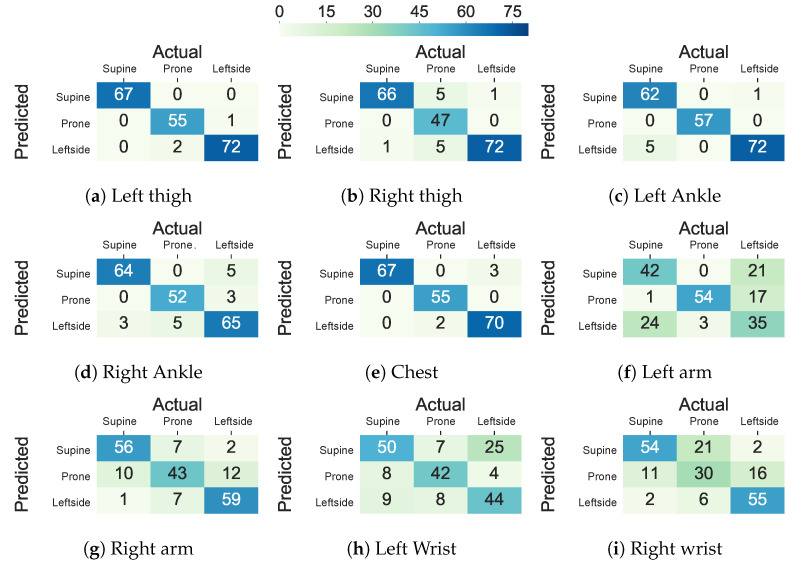
Confusion matrix of the AdaLSTM classifier in classifying lying postures into supine, prone, and left side for the thighs, ankles, chest, arms, and wrists locations using Class-Act dataset.

Figure 7 shows the confusion matrices of the AdaLSTM classifier trained on the data from the thighs, ankles, arms, and wrists using the Class-Act dataset. The models trained on the chest, left thigh, right thigh, left ankle, and right ankle confuse 2.5% 1.5%, 6.1%, 3.0%, and 8.1% of the lying episodes. However, the number of misclassified episodes increases to 33.5%, 19.8%, 30.9%, and 29.4% for the left arm, right arm, left wrist, and right wrist classifiers. We note that the higher misclassification rate when the sensor is on the left arm than the right arm sensor is due to the confusion of the left side and prone postures.

### 5.4. Deep Learning vs. Traditional Machine Learning

In this section, we investigate the possibility of replacing feature-based machine learning models with deep recurrent neural networks (RNNs). For this purpose, we validate the proposed and state-of-the-art deep learning and feature-based classifiers in order to detect four major lying postures from the integrated dataset.

**Figure 8:**
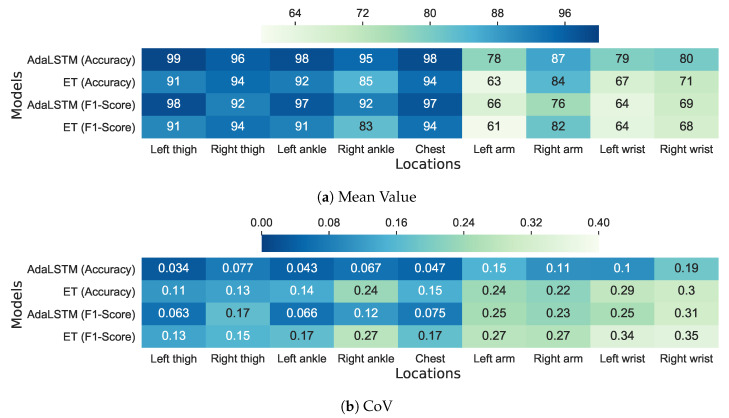
Comparison between the mean and CoV of *F*_1_ score (%) of the ensemble tree and AdaLSTM classification models for nine body locations on the Class-Act dataset using LOSO validation.

Figure 8 compares the mean and CoV of $F_{1}$ score and accuracy metrics between AdaLSTM and ensemble tree classifiers. These classifiers are evaluated while using the Class-Act dataset, including 22 subjects and nine sensor locations on the body. AdaLSTM achieves 2–10% higher accuracy and 3–9% higher $F_{1}$ score than the Ensemble tree classifier when applied to the data collected from the sensor on the chest, the thighs, or the ankles, as shown in Figure 8a. The gap between the performance of the two classifiers increases to 3–15% in accuracy when tested on the data that were collected from the arms or the wrists. Because CoV represents the ratio of variation to the mean of a metric, lower CoV values show a more promising classification performance. AdaLSTM achieves lower CoV values over all the sensor locations, therefore, it adopts a better generalization to cross-subject variations when comparing to the ensemble tree classifier, as shown in Figure 8b. This gap between the performance of the models demonstrates that deep RNNs are more capable of capturing higher-level patterns in noisy data with high variance across subjects, such as the data that were collected from the sensor on the wrists, or the arms.

In addition, we performed Kruskal’s statistical test between the CoV values of the AdaLSTM and ensemble tree classifiers in order to identify any significant difference between the median of the two groups. Kruskal’s test on the CoV of $F_{1}$ score and accuracy show a p-value of 0.100 and 0.006, respectively. These results could not reject the null hypothesis; therefore, they show no significant difference between the performance of the two classifiers. Note that both 0.100, and 0.007 are marginally bigger than the α=0.005, which suggests evaluation on a larger dataset.

### 5.5. Comparison with the State-Of-The-Art

We compare the performance of the proposed models against the state-of-the-art in lying posture detection while using a single accelerometer sensor. The proposed and competing models are described, as follows.

- ET is the proposed feature-based classifier, which is an ensemble of decision trees trained on 48 time-domain features.
- AdaLSTM is the proposed deep learning model, which is an adaptive long short-term memory network with Adam optimizer and decaying learning rate.
- LDA, as proposed by Zhang et al., is a linear discriminate analysis (LDA) classifier trained on the mean value of the signal [11].
- SVM, as proposed by Jeng et al., is a multi-class linear kernel support vector machine classifier trained on the mean value of the tri-axial accelerometer signal [31].
- LSTM is a long short-term memory network with the same structure as the AdaLSTM, but with a fixed learning rate of 0.01.

#### 5.5.1. Class-Act Dataset

Table 4 compares the $F_{1}$ score mean and CoV of the proposed models AdaLSTM and ET against the state-of-the-art deep learning and feature-based models on the Class-Act dataset. The class-act dataset contains data from three major lying postures, including supine, prone, and left side, and nine different sensor locations. Because CoV shows the ratio of variation to mean for a metric, a lower $F_{1}$ score CoV value represents a more promising model. The linear feature-based classifiers including LDA and SVM obtain >88.3% $F_{1}$ scores and <0.26 CoV when applied to data from the thighs, ankles, and chest; however, their performance significantly drops to 50.7–82.2% $F_{1}$ score and 0.22–0.36 CoV when the sensor is moved to the arms or wrists. The competing deep learning model, LSTM, maintains 83.7–92.5% mean $F_{1}$ score for the thighs, ankles, and chest locations, and 51.6–75.5% mean $F_{1}$ score for the arms and wrists location. AdaLSTM outperforms the competing deep learning and feature-based classifiers over all the body locations except for the right thigh, right arm, and left wrist. It achieves 91.5–98.2% mean $F_{1}$ score and 0.06–0.17 CoV for the thighs, ankles, and chest body locations. It shows the most promising result when applied to the sensor on the left thigh with 98.2% $F_{1}$ score and 0.06 CoV. These results demonstrate the power of deep learning and salient of the left thigh in detecting the lying postures for a new subject. We note that neural networks with simpler structures such as LSTM with a fixed learning rate in this paper could not extract useful features and patterns from the raw data automatically from limited data; therefore, choosing the proper parameters for the deep learning models is a crucial factor in their performance.

Figure 6 and Figure 7 show the confusion matrix of the ET and AdaLSTM classifiers for the sensor on the thighs and the wrists locations. Both of the classifiers mislabel a few of the episodes when applied to data from the sensor on the thighs, as shown.

#### 5.5.2. Integrated Dataset

Table 5 shows the mean $F_{1}$ score and CoV values of lying posture detection on the dataset of four major lying postures, including supine, prone, left side, and right side, and five sensor locations, including thighs, wrists, and chest. The results are leave-one-subject-out validated because it is a more realistic validation scenario for the application of human lying posture tracking.

As shown in Table 5, ET and AdaLSTM classifiers achieve a promising range of $F_{1}$ score across all of the body locations (i.e., 63.3% for the right wrist to 97.3% for the chest). ET classifier obtains the highest mean $F_{1}$ score when the sensor is placed on the right thigh (i.e., 97.3%), left wrist (i.e., 65.9%), and right wrist (i.e., 78.6%) locations among all of the algorithms. The linear classifiers, such as LDA [11] and SVM [31] achieve higher $F_{1}$ score than the proposed models in this paper when applied to data that werecollected from the sensor on the left thighs and the chest. The linear relationship between the lying posture and accelerometer readings causes the superiority of state-of-the-art for these locations. On the other hand, extra movements of the hands during lying introduce noise and non-linearity to the data that were collected by the sensor placed on these locations; therefore, the $F_{1}$ score values of linear classifiers drop to 42.1–74.3% for the left and right wrists.

Moreover, the proposed models show lower $F_{1}$ score variation to mean ratio when comparing to the state-of-the-art techniques. Ensemble tree classifier achieves CoV of 0.13 and 0.39, for the right thigh and the left wrist, respectively, and AdaLSTM achieves CoV of 0.12 for the chest. While the CoV of the linear models, including LDA and SVM, increases to the range 0.32–0.50 for the left and right wrist locations. The fact that AdaLSTM was not superior to the ET classifier in all of the scenarios when validated on the integrated dataset could potentially originate from the differences in recording protocols (e.g., sensor positioning) in the new episodes from the DAS dataset when comparing to the results from the previous section.

## 6. Discussion

**Figure 9:**
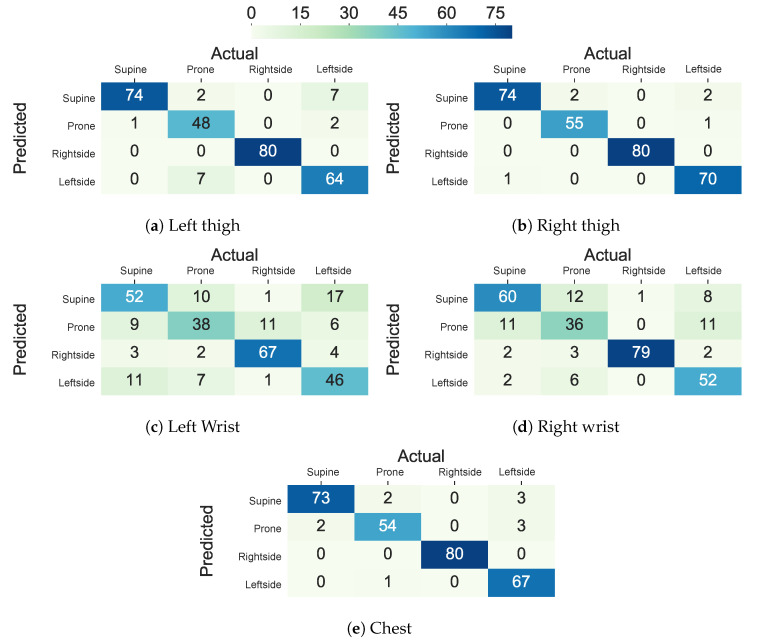
Confusion matrix of ensemble tree classifier in classifying lying postures into supine, prone, and left side for the thighs, ankles, arms, and wrists locations.

We compared the performance of lying posture tracking while using a single accelerometer on nine different body locations in this study. When the ensemble tree classifier is trained on the data that were collected from the sensors on the chest and thighs the lying posture tracking achieves the highest performance and the least cross-subject variations, while classifiers for the wrists and the arms show the least performance and highest within-subject variations. These results demonstrate that individuals might devise arbitrary and dissimilar hand movements during the same lying posture. Figure 9 compares the confusion matrices of the ensemble tree classifiers trained on the data from the chest, thighs, and wrists from the integrated dataset. The chest, left thigh, and the right thigh classifiers confuse 3.8%, 6.6%, and 2.1% of the lying episodes, respectively, while this ratio increases to 28.8% for the left wrist and 20.3% for the right wrist sensor. The confusion between lying postures, such as the supine and prone postures in the wrists’ sensors, is caused by the wrist rotations while lying. In particular, the left side posture is mainly confused with the supine when the sensor is on the left wrist and confused with the supine when the sensor is on the right wrist. Moreover, the majority of the confusions between the left side and prone postures occur when the sensor is on the right wrist, and confusion between the left side and supine posture occurs when the sensor in on the left wrist. These results are mainly due to the similar sensor position during the postures that are confused with each other. For example, the left wrist holds similar positions when the user lays on the back (i.e., supine) and lays on the left side, depending on the rotation of the wrist. Additionally, the right wrist might adopt the same position when the user lays on the front (prone) and lays on the right side.

**Figure 10:**
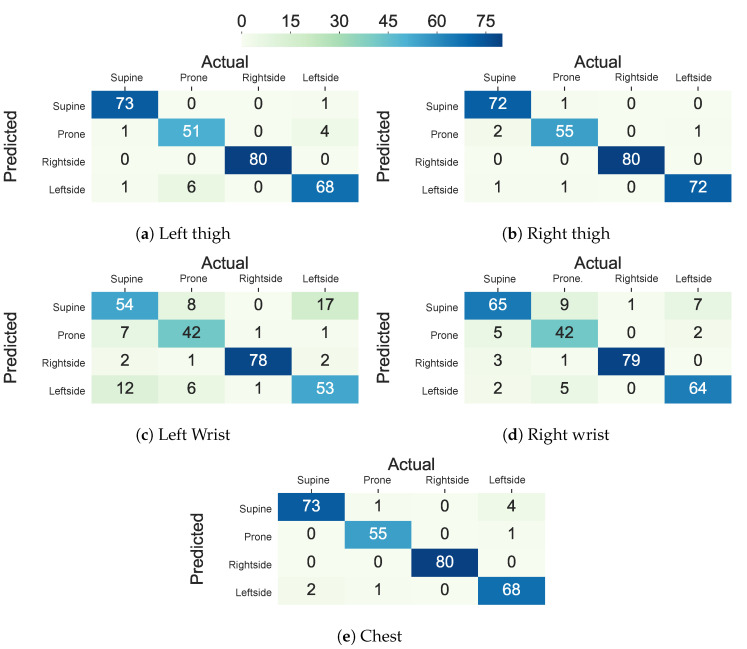
Confusion matrix of AdaLSTM classifier in classifying lying postures into supine, prone, and left side for the thighs, ankles, arms, and wrists locations.

We further investigated the possibility of replacing traditional machine learning with deep learning. Our study showed that deep RNNs such as LSTM can replace the traditional machine learning classifiers as long as adequately designed. Figure 10 shows the confusion matrices for lying posture detection using AdaLSTM on data that were collected from the sensor on the chest, the thighs, and the wrists. These results follow a similar trend as the ensemble tree classifier. 3.1%, 4.2%, and 2.1% of the lying episodes are misclassified when the sensor is worn on the chest, the left thigh, and the right thigh, respectively. While the misclassification rate increases to 20.3% and 12.2% for the left wrist and the right wrist classifiers, respectively. The AdaLSTM confuses 30.0%, and 39.9% less lying episodes when compared to the ensemble tree classifier when the sensor is placed on the left wrist and the right wrist, respectively. These results show the ability of the deep RNNS to capture non-linear relations in the data based on the non-linear operations on a higher level of abstraction. Besides, deep RNNS, such as AdaLSTM, do not require feature-engineering. One major drawback of deep learning is the inability to interpret extracted features through the deeper layers of the network. Moreover, these models are computationally expensive and they require large training datasets to achieve promising results [39].

The fact that end-to-end deep learning neural networks could not improve the performance significantly when comparing to the feature-based classifiers demonstrates the lack of sufficient data as a limitation to this study [22]. We believe adding more data to the training dataset will further improve the performance of AdaLSTM especially for the data from the sensor on the wrists and the arms of the users. We are planning to address this issue in two directions: (1) conduct an extensive multi-modality data collection from a large number of participants performing different lying postures, including main postures and their other variations. Prior research has shown that combining data from different sensor modalities with the appropriate fusion technique will improve the human activity and posture recognition accuracy as integrating independent features will provide a better view of the task. Specifically, deep neural networks could potentially learn more informative features from the data with higher dimension and size [49,50]; and, (2) produce signal-/feature-level synthesis data using data augmentation techniques, such as rotation, permutation, time-wrapping, scaling, magnitude-wrapping, jittering [51], sequence to sequence learning techniques [52], and generative adversarial networks [53].

## 7. Conclusions

We implemented a traditional machine learning classifier, ensemble tree, with time-domain features and a deep recurrent neural network, AdaLSTM, with the decaying learning rate to detect four major lying postures, including supine, prone, left side, and right side, while using a single tri-axial accelerometer sensor. We identified amplitude, mean, minimum, and maximum values of the lateral and vertical axes as the optimal set of time-domain features in traditional machine learning for accurate lying posture tracking while using a single accelerometer sensor. We determined the optimal wearing sites of a single accelerometer sensor (thighs and chest) to accurately detect lying postures. Finally, we evaluated the performance of the proposed models against deep learning and state-of-the-art feature-based lying posture tracking methods while using two publicly available human posture and activity datasets. The proposed AdaLSTM using data from the left thigh and AdaLSTM on the chest locations achieved the highest $F_{1}$ scores (98.2% for the left thigh and 93.5% for the chest) and lowest coefficient of variations (0.06 for the left thigh and 0.07 for the chest) when compared to the other models and sensor locations for the Class-Act dataset. The proposed ensemble tree classifier achieved 97.3% $F_{1}$ score and 0.15 CoV when applied to the data from the sensor on the right thigh, and AdaLSTM obtained 95.0% $F_{1}$ score and 0.12 CoV when applied to the data from the chest sensor from the integration of Class-Act and DAS datasets. These results demonstrated the thighs and chest as the optimum location for accurate lying posture tracking while using a single accelerometer. 

## Figures and Tables

**Table 1:** Extracted time-domain features. $S=(S_{x},S_{y},S_{z})$ is a vector of the tri-axial accelerometer readings, where, $S_{x}$, $S_{y}$, and $S_{z}$ are vectors of accelerometer readings in vertical, lateral, and horizontal directions, respectively. $S_{i}=\left(S_{ix},S_{iy},S_{iz}\right)$ shows a reading of tri-axial accelerometer at timestamp *i*. E(.) represents the expected value of the input variable. Functions min(.),max(.),mean(.),median(.),$tan^{-1}\left(.\right)$, size(.) compute the minimum, maximum, average, median, inverse tangent, and size of an input vector.

Feature	Description	Computation for Signal *S*	Number
AMP	Peak amplitude	max(S)−mean(S)	1–3
MED	Median	median(S)	4–6
MEAN	Mean value	$\mu =\frac{\sum_{i=1}^{N}s_{i}}{N}$	7–9
MAX	Maximum value	max(S)	10–12
MIN	Minimum value	min(S)	13–15
VAR	Variance	$v=\frac{\sum_{i=1}^{N}{\left|s_{i}-\mu \right|}^{2}}{N-1}$	16–18
STD	Standard deviation	$\sigma =\sqrt{\frac{\sum_{i=1}^{N}{\left|s_{i}-\mu \right|}^{2}}{N-1}}$	19–21
RMS	Root mean square	$\frac{\sum_{i=1}^{N}s_{i}^{2}}{N}$	22–24
P2P	Peak to peak	max(S)−min(S)	25–27
ZCR	Zero crossing rate	$\frac{size(\left\{s_{i}|s_{i}==0,i=1,2,..,N\right\})}{N}$	28–30
ENT	Entropy	$-\sum_{i=1}^{N}s_{i}log\left(s_{i}\right)$	31–33
SKN	Skewness	$s=\frac{E{\left(S-\mu \right)}^{3}}{\sigma^{3}}$	34–36
KRT	kurtosis	$k=\frac{E{\left(S-\mu \right)}^{4}}{\sigma^{4}}$	37–39
MAG	Mean Magnitude	$M=\frac{\sum_{i=1}^{N}\sqrt{s_{i}x^{2}+s_{i}y^{2}+s_{i}z^{2}}}{N}$	40
ENG	Energy	$e=\sum_{i=1}^{N}s_{i}^{2}$	41
RNG	Range	r=max(S)−min(S)	42–44
ANG	Angle	$a=max(tan^{-1}\left(\frac{S_{z}}{S_{x}^{2}+S_{y}^{2}}\right))$	45
MAD	Mean absolute deviation	$m=\frac{\sum_{i=1}^{N}\left|s_{i}-\mu \right|}{N}$	46–48

**Table 2:** Performance (%) of the ensemble tree classification in lying-posture detection for nine different body locations on the Class-Act dataset using leave-one-subject-out (LOSO) validation.

Location	*Accuracy*	*Balanced* *Accuracy*	$F_{1}$ Score
Left Thigh	94.5± 6.9	91.3 ± 10.3	90.7 ± 11.8
Right Thigh	96.2 ± 8.1	94.4 ± 12.0	93.5 ± 14.4
Left Ankle	94.9 ± 8.5	92.1 ± 12.8	91.4 ± 15.6
Right Ankle	89.8 ± 13.5	82.9 ± 19.9	82.8 ± 22.7
Chest	96.2 ± 9.1	93.6 ± 13.7	93.6 ± 16.2
Left Arm	78.6 ± 11.7	62.9 ± 15.1	60.9 ± 16.6
Right Arm	89.5 ± 12.1	84.0 ± 18.3	81.6 ± 21.7
Left Wrist	78.6 ± 12.5	67.1 ± 19.1	64.1 ± 21.7
Right Wrist	80.7 ± 14.1	79.7 ± 21.3	67.9 ± 23.8

**Table 3:** Performance (%) of the sequence classification using AdaLSTM in lying-posture detection for nine different body locations on the Class-Act dataset using LOSO validation.

Location	*Accuracy*	*Balanced* *Accuracy*	$F_{1}$ Score
Left Thigh	98.9 ± 8.2	98.4 ± 5.2	98.2 ± 6.2
Right Thigh	95.9 ± 7.3	93.4 ± 11.8	91.5 ± 15.6
Left Ankle	97.9 ± 4.2	96.8 ± 6.3	96.9 ± 6.4
Right Ankle	94.5 ± 6.3	92.4 ± 9.4	91.7 ± 10.7
Chest	98.3 ± 7.1	97.4 ± 7.1	97.3 ± 7.3
Left Arm	77.6 ± 11.7	68.8 ± 14.1	66.3 ± 16.5
Right Arm	86.8 ± 9.2	79.0 ± 14.5	75.7±17.3
Left Wrist	64.8 ± 22.9	64.9 ± 24.8	62.9 ± 23.2
Right Wrist	66.8 ± 26.7	67.6 ± 26.2	66.9 ± 28.9

**Table 4:** Comparison between the mean value and coefficient of variation for $F_{1}$ score of detecting three lying postures for different sensor placements and classifiers including Ensemble Trees (ET), Linear Discriminator Analysis (LDA), LSTM with fixed learning rate (LSTM), and Adaptive LSTM (AdaLSTM) while using Class-Act Dataset. We show the highest $F_{1}$ score value, and lowest CoV metric that models could achieve for each location for LOSO validations in bold.

	Location	ET	LDA	SVM	LSTM	AdaLSTM
**Mean Value (%)**	Left thigh	90.7	95.4	95.4	92.5	**98.2**
	Right thigh	**93.5**	96.1	93.2	84.8	91.5
	Left ankle	92.1	88.3	94.8	90.2	**96.9**
	Right ankle	82.9	90.0	89.5	83.7	**91.7**
	Chest	97.0	94.8	90.1	88.3	**97.3**
	Left arm	60.9	58.1	53.3	53.7	**66.3**
	Right arm	81.6	**82.2**	76.1	75.5	75.7
	Left wrist	**64.1**	55.0	50.7	51.6	64.0
	Right wrist	67.9	65.5	59.2	54.1	**69.4**
**Coefficient of variation**	Left thigh	0.13	0.15	0.13	0.22	**0.06**
	Right thigh	**0.15**	0.16	0.17	0.28	0.17
	Left ankle	0.17	0.18	0.16	0.24	**0.06**
	Right ankle	0.27	0.18	0.12	0.27	**0.11**
	Chest	0.17	0.16	0.26	0.15	**0.07**
	Left arm	0.27	0.33	0.29	0.36	**0.24**
	Right arm	0.26	**0.22**	0.24	0.23	**0.22**
	Left wrist	0.33	0.36	0.36	0.50	**0.25**
	Right wrist	0.35	0.32	**0.29**	0.36	0.31

**Table 5:** Comparison between the mean value and coefficient of variation for $F_{1}$ score of detecting four lying postures for different sensor placements and classifiers including Ensemble Trees (ET), Linear Discriminator Analysis (LDA), Support Vector Machine (SVM), LSTM with fixed learning rate (LSTM), and Adaptive LSTM (Ada-LSTM) for leave-one-subject-out validation using integrated dataset.

	Location	ET	LDA	SVM	LSTM	AdaLSTM
Mean Value(%)	Left thigh	90.6	**94.6**	91.4	92.9	93.7
	Right thigh	**97.3**	96.9	91.4	93.2	94.0
	Chest	95.4	95.4	**96.7**	90.7	95.0
	Left wrist	**65.9**	42.1	58.4	54.1	63.3
	Right wrist	**78.6**	66.7	74.3	42.1	69.2
Coefficient of variation	Left thigh	0.19	0.14	0.25	**0.09**	0.21
	Right thigh	**0.13**	0.15	0.20	0.16	0.17
	Chest	0.13	0.13	0.24	0.25	**0.12**
	Left wrist	**0.39**	0.50	0.50	0.53	0.42
	Right wrist	0.34	0.38	**0.32**	0.40	0.39

## Abbreviations

The following abbreviations are used in this manuscript:

LSTM	Long Short-Term Memory
Bi-LSTM	Bidirectional Long Short-Term Memory
AdaLSTM	Adaptive Long Short-Term Memory
ML	Machine Learning
DL	Deep Learning
RNN	Recurrent Neural Network
DAS	Daily and Sports

## References

[1] Azuh O., Gammon H., Burmeister C., Frega D., Nerenz D., DiGiovine B., Siddiqui A. (2016). Benefits of early active mobility in the medical intensive care unit: A pilot study. Am. J. Med..

[2] Hoyer E.H., Friedman M., Lavezza A., Wagner-Kosmakos K., Lewis-Cherry R., Skolnik J.L., Byers S.P., Atanelov L., Colantuoni E., Brotman D.J. (2016). Promoting mobility and reducing length of stay in hospitalized general medicine patients: A quality-improvement project. J. Hosp. Med..

[3] Neilson J., Avital L., Willock J., Broad N. (2014). Using a national guideline to prevent and manage pressure ulcers. Nurs. Manag..

[4] Kloster R., Engelskjøn T. (1999). Sudden unexpected death in epilepsy (SUDEP): A clinical perspective and a search for risk factors. J. Neurol. Neurosurg. Psychiatry.

[5] Venkateshiah S.B., Collop N.A. (2012). Sleep and sleep disorders in the hospital. Chest.

[6] Dwyer T., Ponsonby A.L., Newman N.M., Gibbons L.E. (1991). Prospective cohort study of prone sleeping position and sudden infant death syndrome. Lancet.

[7] Lee M., Choh A., Demerath E., Knutson K., Duren D., Sherwood R., Sun S., Chumlea W.C., Towne B., Siervogel R. (2009). Sleep disturbance in relation to health-related quality of life in adults: The Fels Longitudinal Study. J. Nutr. Health Aging.

[8] Lindgren M., Unosson M., Fredrikson M., Ek A.C. (2004). Immobility—A major risk factor for development of pressure ulcers among adult hospitalized patients: A prospective study. Scand. J. Caring Sci..

[9] Mador M.J., Kufel T.J., Magalang U.J., Rajesh S., Watwe V., Grant B.J. (2005). Prevalence of positional sleep apnea in patients undergoing polysomnography. Chest.

[10] Wrzus C., Brandmaier A.M., Von Oertzen T., Müller V., Wagner G.G., Riediger M. (2012). A new approach for assessing sleep duration and postures from ambulatory accelerometry. PLoS ONE.

[11] Zhang Z., Yang G.Z. Monitoring cardio-respiratory and posture movements during sleep: What can be achieved by a single motion sensor. Proceedings of the 2015 IEEE 12th International Conference on Wearable and Implantable Body Sensor Networks (BSN).

[12] Kwasnicki R.M., Cross G.W., Geoghegan L., Zhang Z., Reilly P., Darzi A., Yang G.Z., Emery R. (2018). A lightweight sensing platform for monitoring sleep quality and posture: A simulated validation study. Eur. J. Med Res..

[13] Austin D., Beattie Z.T., Riley T., Adami A.M., Hagen C.C., Hayes T.L. Unobtrusive classification of sleep and wakefulness using load cells under the bed. Proceedings of the 2012 Annual International Conference of the IEEE Engineering in Medicine and Biology Society.

[14] Pouyan M.B., Ostadabbas S., Farshbaf M., Yousefi R., Nourani M., Pompeo M. Continuous eight-posture classification for bed-bound patients. Proceedings of the 2013 6th International Conference on Biomedical Engineering and Informatics.

[15] Yousefi R., Ostadabbas S., Faezipour M., Farshbaf M., Nourani M., Tamil L., Pompeo M. Bed posture classification for pressure ulcer prevention. Proceedings of the 2011 Annual International Conference of the IEEE Engineering in Medicine and Biology Society.

[16] Cary D., Collinson R., Sterling M., Briffa K. (2016). Examining the relationship between sleep posture and morning spinal symptoms in the habitual environment using infrared cameras. J. Sleep Disord. Treat. Care.

[17] Lee H.J., Hwang S.H., Lee S.M., Lim Y.G., Park K.S. (2013). Estimation of body postures on bed using unconstrained ECG measurements. IEEE J. Biomed. Health Inform..

[18] Chang K.M., Liu S.H. (2011). Wireless portable electrocardiogram and a tri-axis accelerometer implementation and application on sleep activity monitoring. Telemed. e-Health.

[19] Wai A.A.P., Huang W., Fook V.F.S., Biswas J., Chi-Chun H., Koujuch L. Situation-aware patient monitoring in and around the bed using multimodal sensing intelligence. Proceedings of the 2010 Sixth International Conference on Intelligent Environments.

[20] Huang W., Wai A.A.P., Foo S.F., Biswas J., Hsia C.C., Liou K. Multimodal sleeping posture classification. Proceedings of the 2010 20th International Conference on Pattern Recognition.

[21] Lee J., Hong M., Ryu S. (2015). Sleep monitoring system using kinect sensor. Int. J. Distrib. Sens. Netw..

[22] Goodfellow S.D., Goodwin A., Greer R., Laussen P.C., Mazwi M., Eytan D. (2018). Atrial fibrillation classification using step-by-step machine learning. Biomed. Phys. Eng. Express.

[23] Yongxiang J., Jingle D., Sanpeng D., Yuming Q., Peng W., Zijing W., Tianjiang Z. (2019). Sitting posture recognition by body pressure distribution and airbag regulation strategy based on seat comfort evaluation. J. Eng..

[24] Yang X., Ren X., Chen M., Wang L., Ding Y. (2020). Human Posture Recognition in Intelligent Healthcare. J. Phys. Conf. Ser..

[25] Otoda Y., Mizumoto T., Arakawa Y., Nakajima C., Kohana M., Uenishi M., Yasumoto K. Census: Continuous posture sensing chair for office workers. Proceedings of the 2018 IEEE International Conference on Consumer Electronics (ICCE).

[26] Fallmann S., van Veen R., Chen L., Walker D., Chen F., Pan C. Wearable accelerometer based extended sleep position recognition. Proceedings of the 2017 IEEE 19th International Conference on e-Health Networking, Applications and Services (Healthcom).

[27] Berridge C., Wetle T.F. (2020). Why older adults and their children disagree about in-home surveillance technology, sensors, and tracking. Gerontologist.

[28] Coravos A., Doerr M., Goldsack J., Manta C., Shervey M., Woods B., Wood W.A. (2020). Modernizing and designing evaluation frameworks for connected sensor technologies in medicine. NPJ Digit. Med..

[29] Razjouyan J., Lee H., Parthasarathy S., Mohler J., Sharafkhaneh A., Najafi B. (2017). Improving sleep quality assessment using wearable sensors by including information from postural/sleep position changes and body acceleration: A comparison of chest-worn sensors, wrist actigraphy, and polysomnography. J. Clin. Sleep Med..

[30] Chang L., Lu J., Wang J., Chen X., Fang D., Tang Z., Nurmi P., Wang Z. (2018). SleepGuard: Capturing rich sleep information using smartwatch sensing data. Proc. ACM Interact. Mob. Wearable Ubiquitous Technol..

[31] Jeng P.Y., Wang L.C., Hu C.J., Wu D. A Wrist Sensor Sleep Posture Monitoring System: An Automatic Labeling Approach. https://www.preprints.org/manuscript/201907.0060/v1.

[32] Mannini A., Sabatini A.M. (2010). Machine learning methods for classifying human physical activity from on-body accelerometers. Sensors.

[33] Saeedi R., Schimert B., Ghasemzadeh H. Cost-sensitive feature selection for on-body sensor localization. Proceedings of the 2014 ACM International Joint Conference on Pervasive and Ubiquitous Computing: Adjunct Publication.

[34] Baskin I.I., Marcou G., Horvath D., Varnek A. (2017). Bagging and boosting of classification models. Tutorials Chemoinform..

[35] Breiman L. (2001). Random forests. Mach. Learn..

[36] Fang X., Yuan Z. (2019). Performance enhancing techniques for deep learning models in time series forecasting. Eng. Appl. Artif. Intell..

[37] Lefebvre G., Berlemont S., Mamalet F., Garcia C. (2013). BLSTM-RNN based 3D Gesture Classification. Artificial Neural Networks and Machine Learning—ICANN 2013, Proceedings of the International Conference on Artificial Neural Networks, Sofia, Bulgaria, 10–13 September 2013.

[38] Graves A., Mohamed A.R., Hinton G. Speech recognition with deep recurrent neural networks. Proceedings of the 2013 IEEE International Conference on Acoustics, Speech and Signal Processing.

[39] Gamboa J.C.B. (2017). Deep learning for time-series analysis. arXiv.

[40] Sun S., Xie Z. Bilstm-based models for metaphor detection. Proceedings of the National CCF Conference on Natural Language Processing and Chinese Computing.

[41] Kingma D.P., Ba J. (2014). Adam: A method for stochastic optimization. arXiv.

[42] Olguın D.O., Pentland A.S. Human activity recognition: Accuracy across common locations for wearable sensors. Proceedings of the 2006 10th IEEE International Symposium on Wearable Computers.

[43] Altun K., Barshan B., Tunçel O. (2010). Comparative study on classifying human activities with miniature inertial and magnetic sensors. Pattern Recognit..

[44] Taborri J., Palermo E., Masiello D., Rossi S. Factorization of EMG via muscle synergies in walking task: Evaluation of intra-subject and inter-subject variability. Proceedings of the 2017 IEEE International Instrumentation and Measurement Technology Conference (I2MTC).

[45] Sokolova M., Lapalme G. (2009). A systematic analysis of performance measures for classification tasks. Inf. Process. Manag..

[46] Brodersen K.H., Ong C.S., Stephan K.E., Buhmann J.M. The balanced accuracy and its posterior distribution. Proceedings of the 2010 20th International Conference on Pattern Recognition.

[47] Ronao C.A., Cho S.B. Human activity recognition using smartphone sensors with two-stage continuous hidden Markov models. Proceedings of the 2014 10th International Conference on Natural Computation (ICNC).

[48] Skarpsno E.S., Mork P.J., Nilsen T.I.L., Holtermann A. (2017). Sleep positions and nocturnal body movements based on free-living accelerometer recordings: Association with demographics, lifestyle, and insomnia symptoms. Nat. Sci. Sleep.

[49] Münzner S., Schmidt P., Reiss A., Hanselmann M., Stiefelhagen R., Dürichen R. CNN-based sensor fusion techniques for multimodal human activity recognition. Proceedings of the 2017 ACM International Symposium on Wearable Computers.

[50] Xue Y., Ju Z., Xiang K., Chen J., Liu H. (2018). Multimodal human hand motion sensing and analysis—A review. IEEE Trans. Cogn. Dev. Syst..

[51] Fawaz H.I., Forestier G., Weber J., Idoumghar L., Muller P.A. (2018). Data augmentation using synthetic data for time series classification with deep residual networks. arXiv.

[52] DeVries T., Taylor G.W. (2017). Dataset augmentation in feature space. arXiv.

[53] Wang J., Chen Y., Gu Y., Xiao Y., Pan H. SensoryGANs: An Effective Generative Adversarial Framework for Sensor-based Human Activity Recognition. Proceedings of the 2018 International Joint Conference on Neural Networks (IJCNN).

